# Estimation of pulse wave analysis indices from invasive arterial blood pressure only for a clinical assessment of wave reflection in a 5-day septic animal experiment

**DOI:** 10.1007/s11517-025-03328-8

**Published:** 2025-02-27

**Authors:** Diletta Guberti, Manuela Ferrario, Marta Carrara

**Affiliations:** Department Electronics, Information, and Bioengineering (DEIB), Politecnico Di Milano, Milan, Italy

**Keywords:** Pulse wave analysis, Wave reflections, Transfer function, Blood flow estimation

## Abstract

Wave separation analysis (WSA) is the gold standard to analyze the arterial blood pressure (ABP) waveform, decomposing it into a forward and a reflected wave. It requires ABP and arterial blood flow (ABF) measurement, and ABF is often unavailable in clinical settings. Therefore, methods to estimate ABF from ABP have been proposed, but they are not investigated in critical conditions. In this work, an autoregressive with exogenous input model was proposed as an original method to estimate ABF from the measured ABP. Its performance in assessing WSA indices to characterize the arterial tree was evaluated in critical conditions, i.e., during sepsis. The triangular and the personalized flow approximation and the multi-Gaussian ABP decomposition were compared to the proposed model. The results highlighted how the black-box modeling approach is superior to other flow estimation models when computing WSA indices in septic condition. This approach holds promise for overcoming challenges in clinical settings where ABF data are unavailable.

**Graphical abstract:**

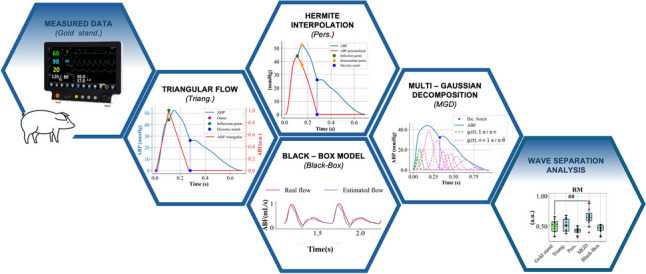

## Introduction

The arterial pulse wave, generated by the left ventricle, propagates from the heart along the circulatory system and is partially reflected due to bifurcations and impedance mismatch typical of the arterial tree; this phenomenon generates multiple reflected waves that travel back towards the heart. Therefore, the reflected waves arise at multiple sites in the arterial tree, where geometry or stiffness changes occur, making the reflection greatly influenced by arterial properties [1]. The arterial waveform measured at any arterial site results from the summation of a forward wave (P_f_), generated by the heart, and a backward (or reflected) wave (P_b_), generated by proximal and distal reflection sites [2]. The time of arrival and magnitude of the reflected wave determine the shape of the arterial blood pressure (ABP) waveform [1] that can be classified as follows: (i) type A, where the reflected wave arrives early during systole and contributes to the rise of systolic pressure opposing ventricular ejection, thereby increasing the cardiac afterload; (ii) type B, where the reflected wave arrives close to the systolic peak; (iii) type C, where the reflected wave arrives after the systolic peak and contributes to increase diastolic pressure, sustaining the myocardial perfusion gradient in the coronary arteries.

Wave separation analysis (WSA) is the gold standard to investigate pulse wave propagation and reflection within the arterial tree, providing insights regarding independent forward and backward wave magnitude and thus contributing towards a quantitative evaluation of cardiovascular risk. For example, reflection magnitude (RM), computed as the ratio between P_b_ and P_f_ amplitudes, has been demonstrated to be strongly predictive of heart failure and adverse cardiovascular events [3]. Furthermore, the amplitude of P_b_ has been proven to be predictive of both cardiovascular events and all-cause mortality in the general population [3, 4]. WSA has been widely employed in chronic patients, e.g., hypertensive patients [5], to monitor the slow structural changes in vascular properties, assessed as alterations in the pulse wave propagation and shape.

Our group has recently shown the potential of WSA in the assessment of cardiovascular alterations in a polymicrobial sepsis model, and how WSA-derived indices can integrate traditional hemodynamic parameters to better evaluate the cardiovascular response to therapies, for example, during sepsis and septic shock [6].

However, WSA necessitates the simultaneous registration of ABP and arterial blood flow (ABF) at the same measurement site. The measurement of ABF is typically invasive, i.e., it requires the placement of an ultrasonic flow probe around the vessel, specific types of equipment and skilled operators, and therefore is not commonly available. Non-invasive techniques, like echo Doppler, require expert operators, and echo imagining is not usually performed for this purpose and not frequently in intensive care unit (ICU).

To address the limited availability of ABF measures, several methods have been investigated to perform pulse wave analysis when only arterial blood pressure is available.

Westerhof and colleagues [7] first proposed the triangular method, which leverages the fiducial points on ABP waveform to generate an uncalibrated flow waveform assumed to have a triangular shape. Despite being widely used, this method poorly approximates the measured ABF waveform, potentially leading to errors in WSA [8]. Kips and colleagues [9] proposed a more physiological shape for the estimate of ABF waveform by averaging and normalizing measurements of flow waveforms from 74 participants of the Asklepios population study. This method has provided a better estimate of the RM index than the triangular flow waveform, but the main limitation is that the averaged flow waveform may not be representative of subjects with age or clinical characteristics far beyond from those of the Asklepios population. Recently, modifications to the triangular method have been proposed to allow a more physiological shape of the ABF waveform estimate by adopting the Hermite interpolation, although the results are obtained from simulated data or healthy participants only [10]. A different approach proposed in [11] employs a multi-Gaussian decomposition (MGD) technique in order to directly estimate backward and forward arterial waveforms without the estimate of ABF; the methods consist of the decomposition of ABP into its distinct backward and forward components through the summation of Gaussian functions that most effectively approximate the ABP waveform.

The main drawback associated with all the above-mentioned methods is that the flow estimation depends on the identification of fiducial points on the ABP pulse waveform, i.e., the inflection point and the dicrotic notch. The identification of these fiducial points is algorithm-based, and this can potentially introduce further bias in the ABF estimation. Moreover, in the case of healthy or stable conditions, the fiducial points are plain on ABP waveforms, but they may become difficult to detect on pulse waveforms that are morphologically altered.

In this study, we propose a method for blood flow estimate from an invasive ABP measure based on a black-box modeling approach in order to obtain reliable PWA indices from signals collected during a 5-day swine experiment investigating sepsis and septic shock. We adapted the method proposed by Arai et al. [12], where aortic ABP and ABF were used instead of carotid registrations as in our study. Carotid ABF is not equal to 0 during the diastolic phase, different from aortic ABF. However, carotid blood pressure is considered a well-established surrogate of central blood pressure, and it is frequently used as a surrogate of aortic blood pressure measurement [13].

The objective of the present study is, therefore, to evaluate the estimation of the PWA indices obtained by the proposed approach in comparisons to the gold standard, i.e., the indexes obtained directly by using the measurements of ABF, and to verify if it could overcome the limitations of the most common methods, i.e., the triangular flow method [7], the personalized flow estimation [10], and the multi-Gaussians decomposition [11].

In particular, our goal is to evaluate the reliability and efficacy of these methods for WSA indices assessment in non-physiological and unstable conditions, where important morphological changes of ABP waveform challenge the reliable estimation of arterial blood flow and, therefore, of arterial tree characteristics derived with WSA techniques.

## Materials and methods

### Experimental protocol and instrumentation

This study was carried out in collaboration with the Inselspital University Hospital of Bern, Switzerland. The animal protocol was performed in accordance with the EU Directive 2010/63/EU for animal experiments and the ARRIVE guidelines for animal research and with the approval of the Animal Care Committee of the Canton of Bern (BE103/16). The experiment was designed in accordance with the Surviving Sepsis Campaign Guidelines; indeed, the animal model exhibits characteristics consistent with the clinical phenotype of sepsis, including hemodynamic variables, inflammatory cytokine patterns, and acute kidney injury despite increased renal plasma flow, as previously reported [14, 15].

Twenty domestic pigs (weight: 39.8 ± 2.7 kg; male/female: 1:1) were originally used for the experiment. Details about surgical preparation and instrumentation are reported in previous works [14]. After instrumentation, 1-h stabilization was allowed, followed by baseline measures (time point T1). Then, the pigs were randomized to sepsis (SS, *n* = 10) or sham (SH, *n* = 10). Sepsis was induced by peritoneal instillation of 2 g/kg of autologous feces dissolved in 250 mL warmed glucose 5% solution. After 8 h of observation without resuscitation, time point T2 was defined, referring to the septic condition. Then, protocol-based resuscitation was started and continued for 3 days (resuscitation period, RP), including fluid infusion (e.g., ringer lactate), vasopressor support (e.g., noradrenaline), electrolyte maintenance, antibiotic therapy, and reduction of body temperature. Details about resuscitation maneuvers can be found in [15]. ABP and ABF signals were evaluated just before 24 h (T3), 48 h (T4), and 74 h after resuscitation (T5). At the end of the experiment, animals were euthanized by bolus infusion of 40 mmol of potassium chloride.

An arterial catheter (5F, Cordis AVANTI, Fremont, CA, USA) was placed in the left carotid artery to continuously register carotid ABP, and a flow probe (Transonic Systems Inc., Ithaca, NY, USA) was placed around the right carotid artery to continuously acquire ABF. The signals were recorded with a sampling frequency of 100 Hz by a data acquisition system (Soleasy™; National Instruments Corp., Austin, TX, USA).

### Data pre-processing

**Fig. 1:**
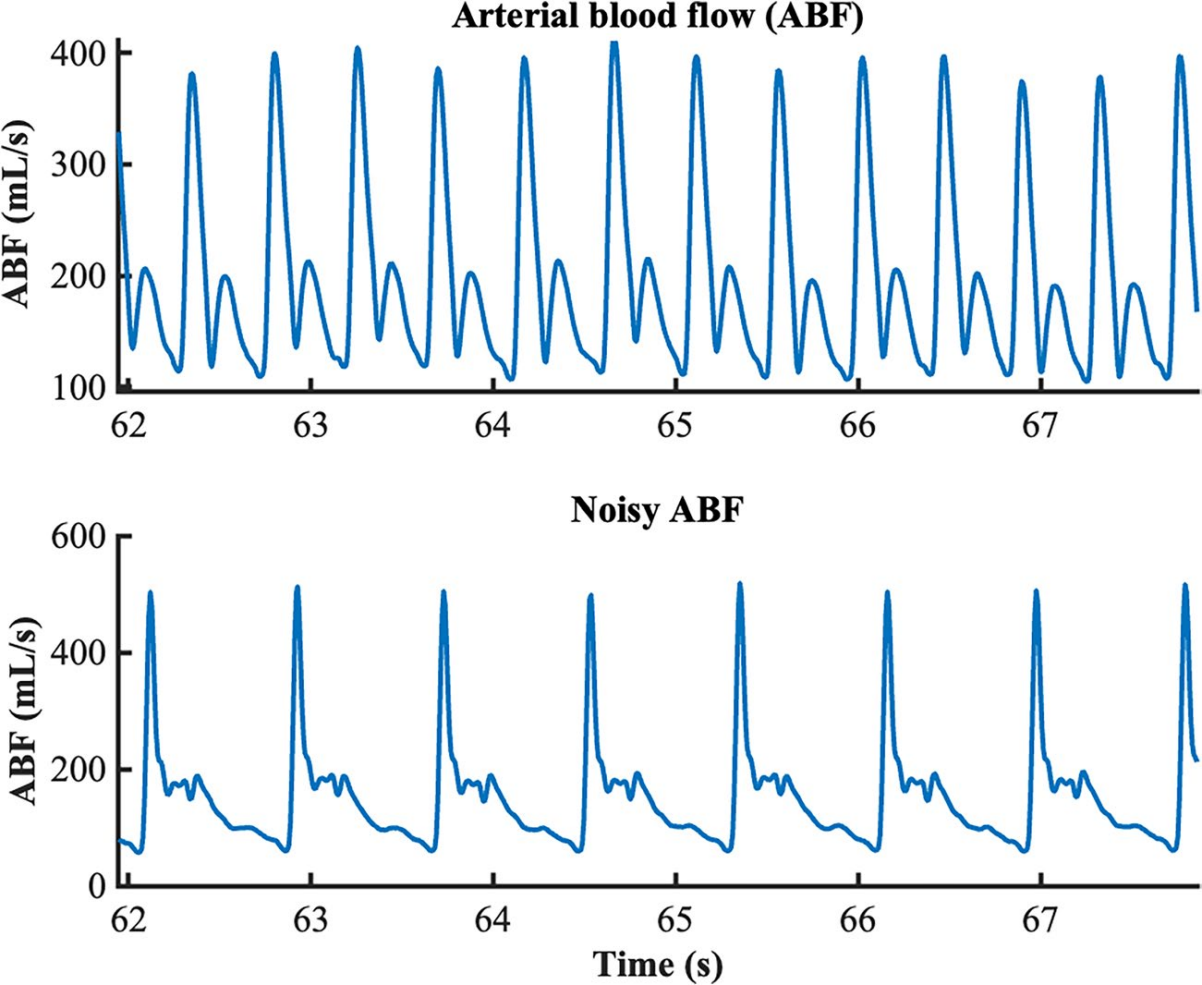
Top panel: example of good quality arterial blood flow (ABF). Bottom panel: example of ABF waveform affected by noise that prevents the signal from being used for further analyses

As illustrated herein, the pre-processing analyses constitute the initial phase preceding the subsequent analyses intended for the extraction of indices and ABF estimation. Portions of 5–10 min of signals were selected from arterial blood pressure and flow waveforms at each time point for successive analyses. These segments were supposed to be stationary, artifacts-free, and far from possible maneuvers (e.g., drug infusion, nursing procedures, catheter flushing), which can affect signal quality and stationarity. Six out of 20 animals had to be excluded from the study due to the very low quality of signals. The final dataset, thus, consists of 14 pigs: 5 for the sham group and 9 for the septic group (see Table 1). Moreover, certain time points from some pigs were excluded for the same reasons, resulting in a final dataset of 50 ABP and ABF pairs of signals. This is a long experiment; catheters and sensor are periodically checked and flushed, but it may happen to still have noisy pieces of recordings. An example of a low-quality signal excluded from the study is reported in Fig. 1.

**Table 1:** Number of animals included in the study for each time point of the experiment, given that 6 out 20 animals were excluded as the ABF signals were of poor quality for technical reasons

	T1	T2	T3	T4	T5
Number of pigs included	14/14	14/14	12/14	10/14	0/14

All the signals were first filtered with a low pass moving average filter (order 2–5) to eliminate high-frequency noise. The onsets of each cardiac beat were then identified using the algorithm proposed by Zong and colleagues [16]. Briefly, the algorithm employs a windowed and weighted slope sum function (SSF) to extract ABP or ABF waveform features. Adaptive thresholding and search strategies are applied to the SSF signal to detect signal pulses and to determine their onsets.

**Fig. 2:**
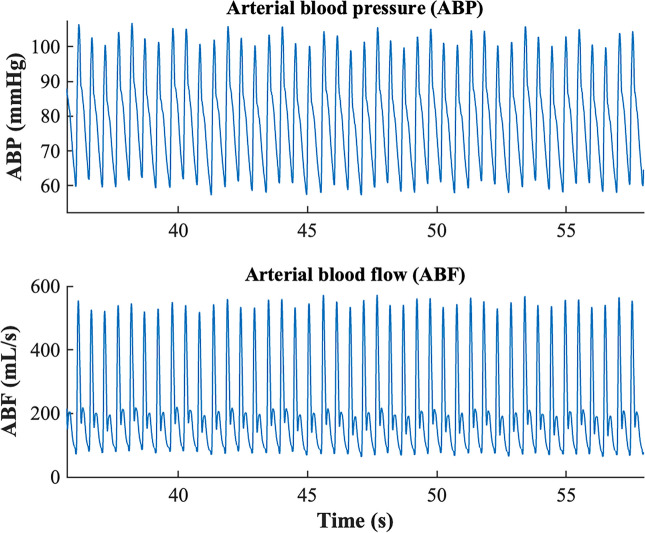
Example of arterial blood pressure (ABP) trace (top panel) and arterial blood flow (ABF) trace (bottom panel). It is possible to observe how beats are similar in terms of waveform morphology for the entire portion of signal

Successively, 10 consecutive beats were selected for each signal at each time point. ABP and ABF signals were aligned as a few samples of delay may occur due to the different measurement sites. Finally, a beat template was obtained by averaging the 10 beats. The rationale of using 10 beats is based on the need to have enough beats for a reliable template estimation and a reasonable amount of data for the black-box model identification. Selecting the stationary portion of signals, the shape of ABP and ABF waveforms remains pretty similar in terms of morphological characteristics for large portions of signals, as shown in the example of Fig. 2.

In this study, ABP waveforms were grouped according to the different beat morphology, i.e., type A or type B beats, irrespective of the time point. Type C beats were included in type B groups as they were very few and they have a similar physiological meaning. This classification was done based on the occurrence time of the inflection point, according to the theoretical framework: type A if the inflection point occurs before the systolic peak; type B if it occurs in correspondence of the systolic peak or right after. The final dataset was composed of 41 type A beat templates and 9 type B beat templates. Type A waveforms were observed in sham pigs and mainly at baseline and after resuscitation in most SS pigs; type B waveforms were observed instead during the un-resuscitated septic condition [6]. To increase the sample size of the type B group, 10 consecutive beats were selected three times within the same recordings, far from each other. In this way, the final dataset consists of 27 type B templates.

The fiducial points, such as the inflection point *p1* and the dicrotic notch, were identified on ABP templates by means of the algorithm proposed by Karamanoglu [17], which is based on signal derivatives of different orders. After the automatic detection, the correct position of the fiducial points was manually verified and corrected, if necessary, in order to improve ABP waveform classification. To the best of our knowledge, no automatic classification algorithm is available for this purpose.

Finally, the offset value was removed from both ABP and ABF templates, and the amplitude of the ABF template was normalized as its absolute amplitude does not influence the successive analyses [7].

Forward and backward pressure waves were assessed using the beat templates obtained from the measured ABP and ABF as follows [7]:

1 $${P}_{f}=\frac{ABP+{Z}_{c}*ABF}{2}$$

2 $${P}_{b}=\frac{ABP-{Z}_{c}*ABF}{2}$$

where Z_*c*_ represents the characteristic impedance, a local vessel’s parameter that depends on vessel size, wall stiffness, i.e., compliance, and blood properties, and it is independent from wave reflection. Z_*c*_ can be estimated from the input impedance (Z_in_) modulus in the frequency domain as [18]:

3 $${Z}_{c}=\frac{1}{j-i+1}\sum_{k=i}^{j}\left|{Z}_{in, k}\right| \; where \; {Z}_{in, k}=\frac{{ABP}_{k}}{{ABF}_{k}}$$

with ABP_k_ and ABF_k_ being the pressure and flow modulus of the k-*th* harmonic. In this study, we adopted* i* = 2 and *j* = 8 for type A waveforms and *j* = 5 for type B waveforms due to their simpler and smoother ABP morphology so as to be described with fewer coefficients; in this case, averaging higher harmonics components of *Z*_*in*_ would add noise in the estimation [6].

The amount of wave reflection can be assessed by computing the RM and reflection index (RI) defined as follows [7]:

4 $$RM=|{P}_{b}|/|{P}_{f}|$$

5 $$RI=|{P}_{b}|/(\left|{P}_{b}\right|+\left|{P}_{f}\right|)$$

where $|{P}_{b}|$ and $|{P}_{f}|$ are the amplitude, i.e., the difference between the maximum and the minimum value, of backward and forward waves, respectively. The amplitude of P_b_ can also be used to quantify the amount of reflection in the system.

### Triangular flow approximation

**Fig. 3:**
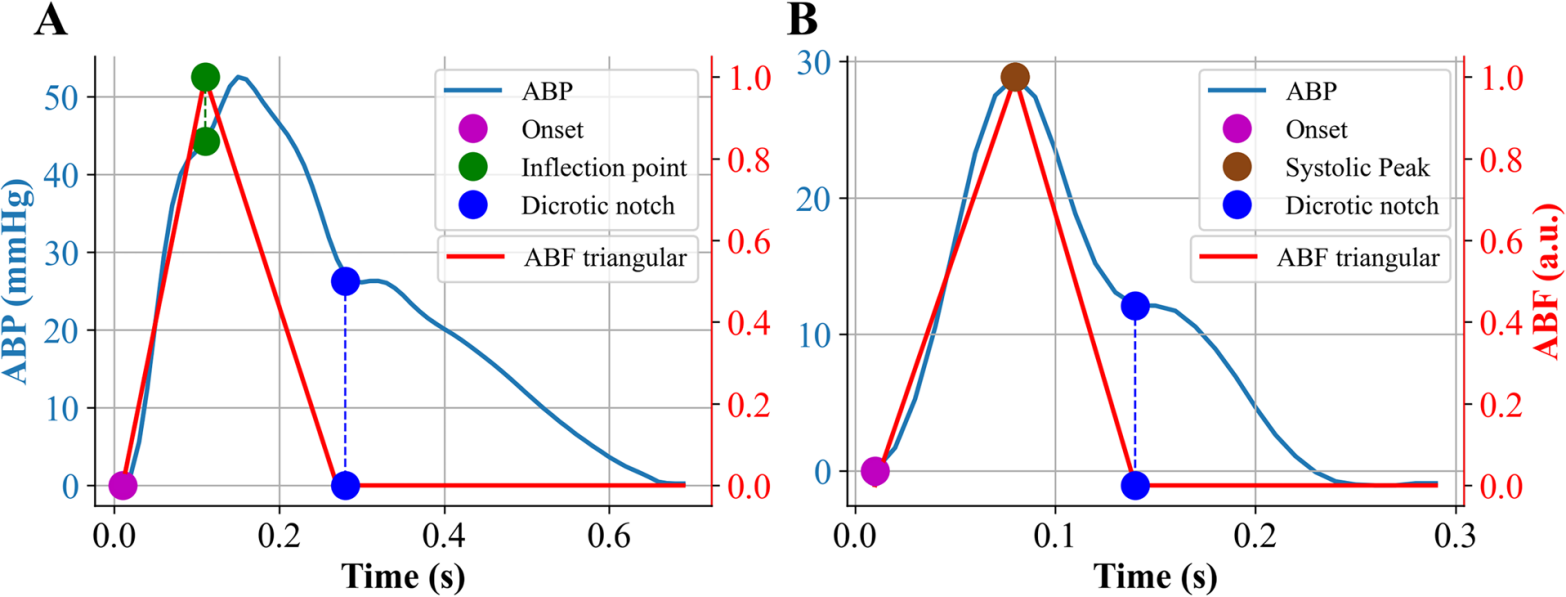
Example of triangular approximation of arterial blood flow (ABF) based on arterial blood pressure (ABP) fiducial points. **A** Type A waveform; **B** Type B waveform

Triangular flow approximation was computed using the ABP beat templates. The method is based on specific ABP fiducial points, as proposed in the original publication [7]. In particular, for type A waveforms, the onset of the ABP pulse represents the start of the triangular wave; before this point, ABF is assumed to be 0. The inflection point *p1* marks the peak of the triangular flow wave, i.e., *p1* corresponds to the peak flow. The dicrotic notch identifies the end of the triangular wave after which ABF is set to 0 (Fig. 3A).

For type B waveforms, the algorithm was slightly modified so that the triangular flow peak is located in correspondence of systolic peak, according to pressure-flow physiological relationships (Fig. 3B). The absolute amplitude of approximated ABF does not influence the estimation of forward and reflected components as reported in [7]; therefore, the peak amplitude was set to 1. Forward and backward pressure waves were estimated according to Eqs. 1 and 2, and they were successively used to estimate RM and RI.

### Personalized flow estimation

Similarly to the triangular flow wave, this method needs to identify three reference points on the ABP waveform [10]: (1) the inflection point, *p1*; (2) an intermediate point; (3) the dicrotic notch. The intermediate point is defined in correspondence of systolic blood pressure (SBP) occurrence with a value equal to $(MAP+SBP)/2$, where SBP represents the maximum value within the beat, and MAP is the mean arterial pressure, determined as the average of all the pressure values within the beat. The Hermite interpolation function [19] was then applied to create a more personalized ABF approximation, as proposed in the original study [10].

The three points are required to satisfy the Hermite interpolation function condition. To achieve a smooth and continuous curve on the generic interval [a, b], a segmented Hermite interpolation is employed. Given the nodes $a\le {x}_{0}<{x}_{1}<\dots <{x}_{n}\le b$, the function and the derivative value of each node are the following ${y}_{i}=f\left({x}_{i}\right) \; \text{and} \; {{y}_{i}}^{\prime}={f}^{\prime}\left({x}_{i}\right), \; i=\text{1,} \; \text{2},\; \dots \; n$. A piecewise cubic interpolation polynomial ${H}_{3}\left(x\right)$ is constructed on [a, b]. This polynomial satisfies the following interpolation conditions [10] ${H}_{3}\left({x}_{i}\right)= {y}_{i},\; {{H}^{\prime}}_{3}\left({x}_{i}\right)={{y}^{\prime}}_{i}, \; i=\text{1,} \; \text{2},\; \dots \; n$. ${H}_{3}\left(x\right)$ on the interval [${x}_{i-1}$, ${x}_{i}$] is computed as follows:

6 $${H}_{3}\left(x\right)=\frac{1}{{h}_{i}^{2}}\left[\left(1+2\frac{x-{x}_{i-1}}{{h}_{i}}\right){\left(x-{x}_{i}\right)}^{2}{y}_{i-1}+\left(1-2\frac{x-{x}_{i-1}}{{h}_{i}}\right){\left(x-{x}_{i-1}\right)}^{2}{y}_{i}+\left(x-{x}_{i-1}\right){\left(x-{x}_{i}\right)}^{2}{y}_{i-1}^{\prime}+{\left(x-{x}_{i-1}\right)}^{2}\left(x-{x}_{i}\right){y}_{i}^{\prime}\right]$$

where $$x\in [{x}_{i-1},\; {x}_{i}]$$ and $${h}_{i}={x}_{i}-{x}_{i-1}$$.

**Fig. 4:**
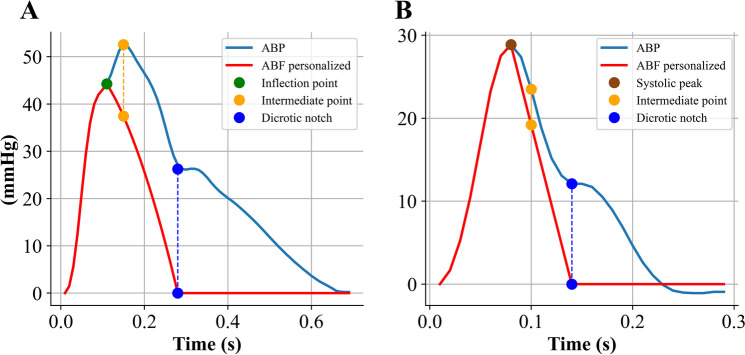
Example of personalized arterial blood flow (ABF) approximation based on the Hermite interpolation function. ABP, arterial blood pressure. **A** Type A waveform; **B** Type B waveform

The initial part of ABF was set equal to the rising portion of the ABP waveform until *p1*, and then the cubic Hermite interpolation function was employed to approximate ABF from *p1* to the dicrotic notch of ABP waveform; after the dicrotic notch, ABF was assumed to be 0 (Fig. 4A).

**Fig. 5:**
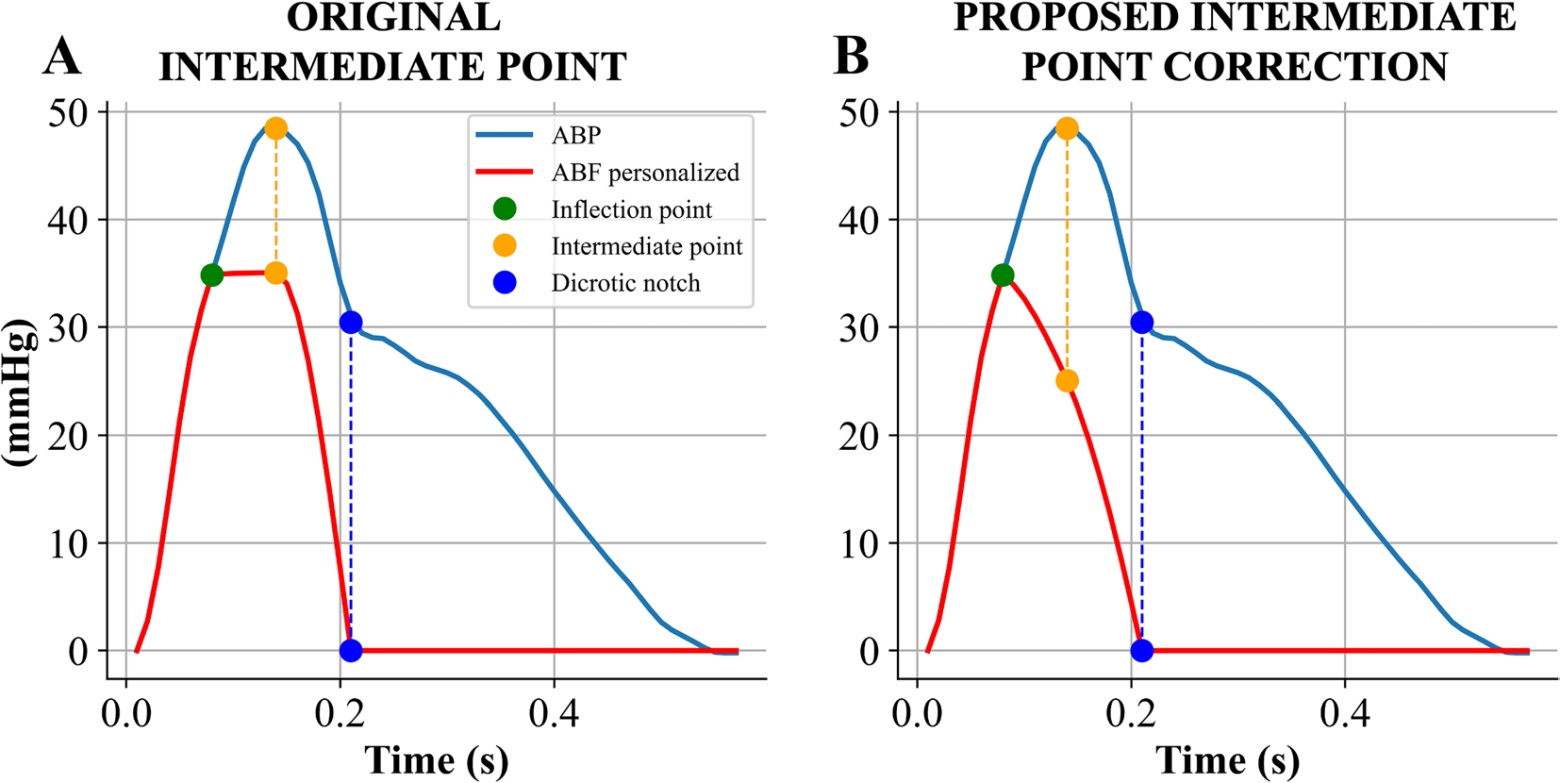
Example of the personalized arterial blood flow (ABF) approximation with an adapted estimation of intermediate point. **A** Type A waveform where the standard intermediate point has a value equal to the inflection point. **B** Intermediate point estimated with the proposed correction for the physiological ABF approximation

The original method was adapted to be applied also in the case of type B waveforms where SBP is used to set the peak of blood flow, i.e., in the absence of *p1* point. In this case, the three reference points are (1) SBP, (2) an intermediate point, and (3) the dicrotic notch. The new intermediate point was placed 2 samples after SBP with an amplitude equal to $(MAP+SBP)/2$; if the obtained value was equal or higher than SBP, it was adjusted to be lower than the SBP by at least 5 mmHg (Fig. 4B). Similarly, in type A waveforms, if the value of the calculated intermediate point exhibited values equal or higher than *p1*, a similar correction was undertaken (Fig. 5).

As for the triangular ABF approximation, the peak amplitude of the personalized ABF approximation was set to 1. From the estimated ABF, forward and backward pressure waves were computed, and the indices RM and RI were assessed.

### Multi-Gaussian decomposition

The MGD consists of decomposing the ABP template utilizing weighted and shifted Gaussian functions, which are then summed to derive the forward and backward components [11]. In details, ${G}_{1}\left(t\right)$ and ${G}_{2}\left(t\right)$ are computed as the sum of Gaussians ${g}_{i}(t)$:

7 $${G}_{1}\left(t\right)= \sum_{i=1}^{n}{g}_{i}(t)$$

8 $${G}_{2}\left(t\right)=\sum_{i=n+1}^{N}{g}_{i}(t)$$

**Fig. 6:**
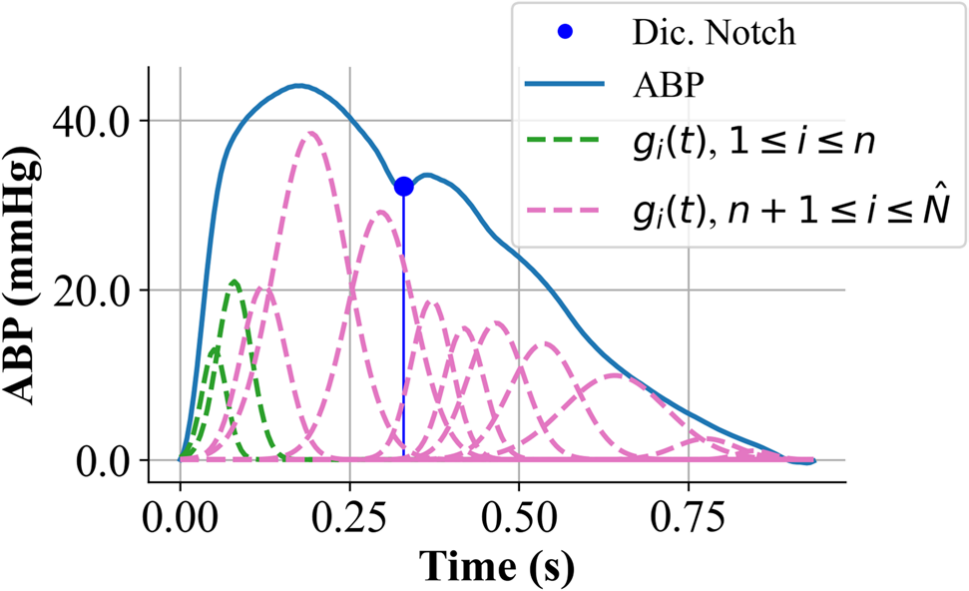
Example of multi-Gaussian ABP decomposition. Blue line is the measured arterial blood pressure (ABP); green dashed lines are the Gaussians (${g}_{i}(t$)) used to determine ${G}_{1}\left(t\right)$; pink dashed lines are the ones used to determine ${G}_{2}\left(t\right).$

Here, *N* represents the total number of Gaussians, and *n* corresponds to the number of Gaussians that extinguish before the dicrotic notch. In this study, the sampling frequency is 100 Hz, so the signal was resampled to a higher frequency of 200 Hz to increase the number of samples for Gaussian estimation. Each Gaussian ${g}_{i}(t)$ is characterized by the mean μ, the amplitude* a*, and the standard deviation σ; these parameters were found iteratively using a non-linear least square minimization. Initially, to identify the optimal number of Gaussians $\widehat{N}$, the parameters were computed for every *N* ranging from 6 to 15. The ABP template was divided into *N* segments; the initial *μ*_*i*_ and amplitude *a*_*i*_ were set equal to the maximum ABP value within each segment, and σ_*i*_ was set to 0.01. $\widehat{N}$ was then identified by minimizing the residual norm between the ABP curve and the curve $\sum_{i=1}^{N}{g}_{i}(t)$. In conclusion, the result of this optimization process includes $\widehat{N}$, the optimal number of Gaussians, and the values of *μ*, σ, and *a* for each Gaussian. The parameter *n* was then determined based on the value distribution of the first functions ${g}_{i}(t)$, i.e., the distance between the peak of ${g}_{i}(t)$ and the dicrotic notch must be less than 3σ_i_, typically *n* ranged from 2 to 4. An example of ABP MGD is illustrated in Fig. 6.

*P*_*b*_ and *P*_*f*_ can be computed as a function of ${G}_{1}\left(t\right)$ and ${G}_{2}\left(t\right)$, using the following equations [11]:

9 $${P}_{b}=\frac{{G}_{2}(t)}{2}$$

10 $${P}_{f}={G}_{1}\left(t\right)+{P}_{b}.$$

Similarly to triangular and personalized flow wave approximation, also the MGD method was applied to ABP average templates. RM and RI were then computed using Eqs. 4 and 5.

### Black-box modeling—ARX model

**Fig. 7:**
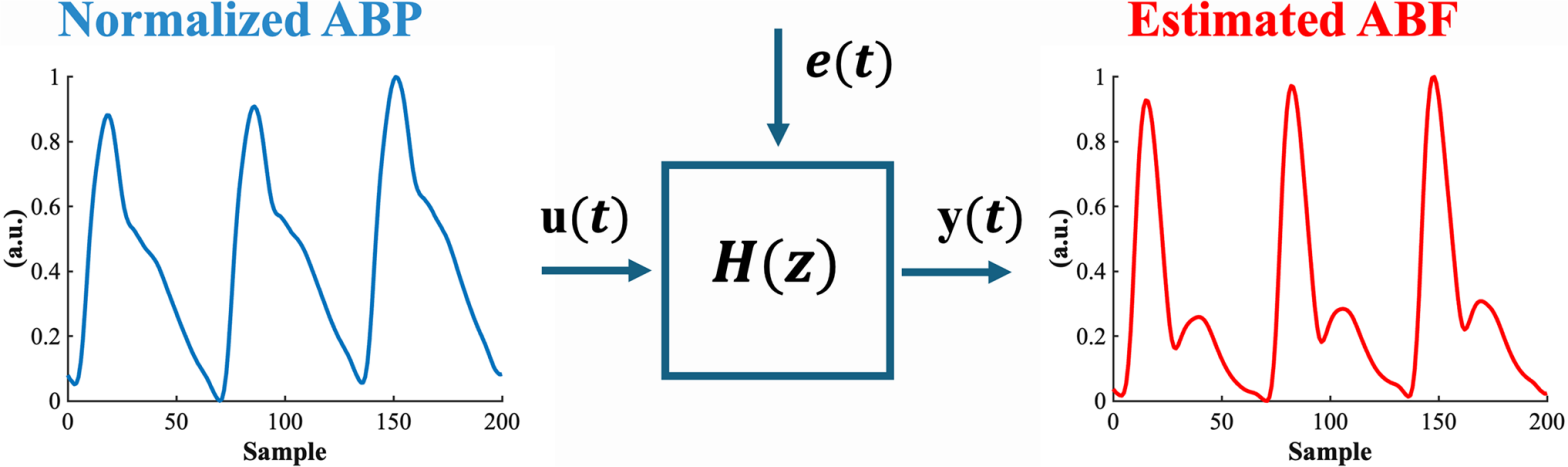
Schematic representation of the black-box model. The input $u(t)$ is the normalized arterial blood pressure (ABP) signal; the output $y(t)$ is the estimated arterial blood flow (ABF); $e\left(t\right)$ is a random noise

An autoregressive with exogenous input (ARX) model for ABF estimation was applied to the 10 consecutive beats selected at each time point. The ABP and ABF signals were both rescaled to range between 0 and 1. As previously stated, we do not need a calibrated ABF signal, and the normalization allows the model to not depend on the ABP amplitude, but only on its morphology. A schematic representation of the model is illustrated in Fig. 7.

Two different models were created according to the type of waveforms (type A or type B), given the highly different morphology of pressure and flow waveforms in these two cases.

Each dataset was divided into 70% train and 30% test set. The coefficients of an autoregressive with exogenous input model were computed on the training set of ABP and ABF signals by standard least-square minimization procedure:

11 $$ABF\left(n\right)={\sum }_{j=1}^{L}{a}_{j}ABF\left(n-j\right)+{\sum }_{j=0}^{P}{b}_{j}ABP\left(n-j\right)+e(n)$$

The orders $L$ and $P$ of the model were selected by using both the Akaike’s Information Criterion (AIC) [20] and the Minimum Description Length (MDL) criterion for both type A and B waveform models for comparison purposes.

For each couple of signals ABP and ABF a model is obtained, and different strategies have been explored to define the final model to be validated on the test set.

*Model 1* and *2* refers to the following procedure: the range for the model orders $L$ and $P$ was set to vary from 1 to 20, and each couple of signals ABP and ABF provides a model with ${K}_{i}$ number of estimated coefficients. The number of coefficients was set equal to the average number $K=1/N\sum_{i}{K}_{i}$, and in the second case, the number of coefficients $K$ was set to the most frequent one, i.e., the statistical mode of ${K}_{i}$. Then, the final model was built by averaging the first *K* coefficients obtained from the different models. *Model 3* and *4*, instead, refers to the following procedure: the range for the model order was set to vary from 1 to 20 in the first case and from 1 to 30 in the second case. Each couple of signals ABP and ABF provides a model with ${K}_{i}$ number of estimated coefficients, and the number of coefficients was kept equal to 20 or 30, respectively. When the model order was found to be ${K}_{i}<20$ or 30, the coefficients ${a}_{j}$ and ${b}_{j}$, with $j=\left({K}_{i}+1\right)\div 20$ or 30, were set to be 0. Then, the final model was built by averaging the 20 or the 30 coefficients thus obtained.

The estimated models were subsequently validated on the test set, and the ABF signals estimated from the measured ABP signals were compared to the measured ABF signals, used as reference, in order to evaluate the models’ predictions. The root mean square error (RMSE) was used to evaluate the goodness of fit of the model:

12 $$RMSE=\sqrt{{~}^{\sum_{i=1}^{N}||y\left(i\right)-\widehat{y}(i){||}^{2}}\!\left/ \!{~}_{N}\right.}$$

where $N$ is the number of data points, $y(i)$ is the i-*th* value of the measured ABF, and $\widehat{y}(i)$ is the corresponding predicted value from the model. The RMSE values obtained using AIC as the optimization criterion for the model order were found to be lower compared to the values obtained using the MDL criterion; therefore, we chose the models optimized using AIC.

The modeled flow signal was used to create an average beat template of the estimated ABF both for type A and B waveforms, as previously described, and then it was employed to compute forward and backward waves, together with RM and RI.

### Statistical analyses

The performance of all the above-mentioned methods for the estimation of PWA indices was compared with the gold standard, i.e., the indexes obtained directly by using the measure of ABF. In particular, the comparisons among all the methods were performed on the signals of the test set only, i.e., in signals not already used to train the model.

Mann–Whitney U (or Wilcoxon rank-sum) test was used to verify significant differences in the indices between the gold standard values, i.e., the ones resulted by using measured pressure and flow signals, and each of the four methods. Significance was considered with a *p*-value < 0.05. Bland–Altman was used to evaluate the agreement between index values obtained by the different methods compared to the gold standard values.

## Results

Table 2 reports the performances of the estimated models obtained by using both optimization criteria, AIC and MDL, and different approaches to define the coefficient values for the final models, as described in the method section.

**Table 2:** Comparison of root mean square error (RMSE) values obtained using different approaches. Values are reported as median (25th, 75th percentile). L and P are the model orders of the ARX model; *AIC*, Akaike’s Information Criteria; *MDL*, Minimum Description Length (MDL)

	Type A		Type B	
	AIC	MDL	AIC	MDL
*Model 1*	*P* = 12; *L* = 14	*P* = 8; *L* = 10	*P* = 11; *L* = 10	*P* = 9; *L* = 8
RMSE	0.296 (0.292, 0.308)	0.266 (0.227, 0.298)	0.258 (0.185, 0.356)	0.227 (0.158, 0.322)
*Model 2*	*P* = 20; *L* = 8	*P* = 4; *L* = 7	*P* = 20; *L* = 9	*P* = 1; *L* = 9
RMSE	0.396 (0.381, 0.412)	0.290 (0.266, 0.310)	0.380 (0.350, 0.394)	0.428 (0.398, 0.451)
*Model 3*	Average of 20 coefficients			
RMSE	0.084 (0.075, 0.099)	0.093 (0.080, 0.115)	0.080 (0.063, 0.097)	0.077 (0.055, 0.095)
*Model 4*	Average of 30 coefficients			
RMSE	0.097 (0.091, 0.104)	0.118 (0.098, 0.123)	0.086 (0.072, 0.102)	0.080 (0.072, 0.091)

It must be noticed that, as expected, the MDL criterion is more conservative and provides a less complex model. We decided to use the approach with a fixed number of 20 coefficients and the AIC criterion for both type A and type B waveforms as this approach provides better results, and no significant differences were obtained between the AIC and MDL criterion for *model 3*. The error in estimating ABF was, on average, around 8% for both waveform types.

**Fig. 8:**
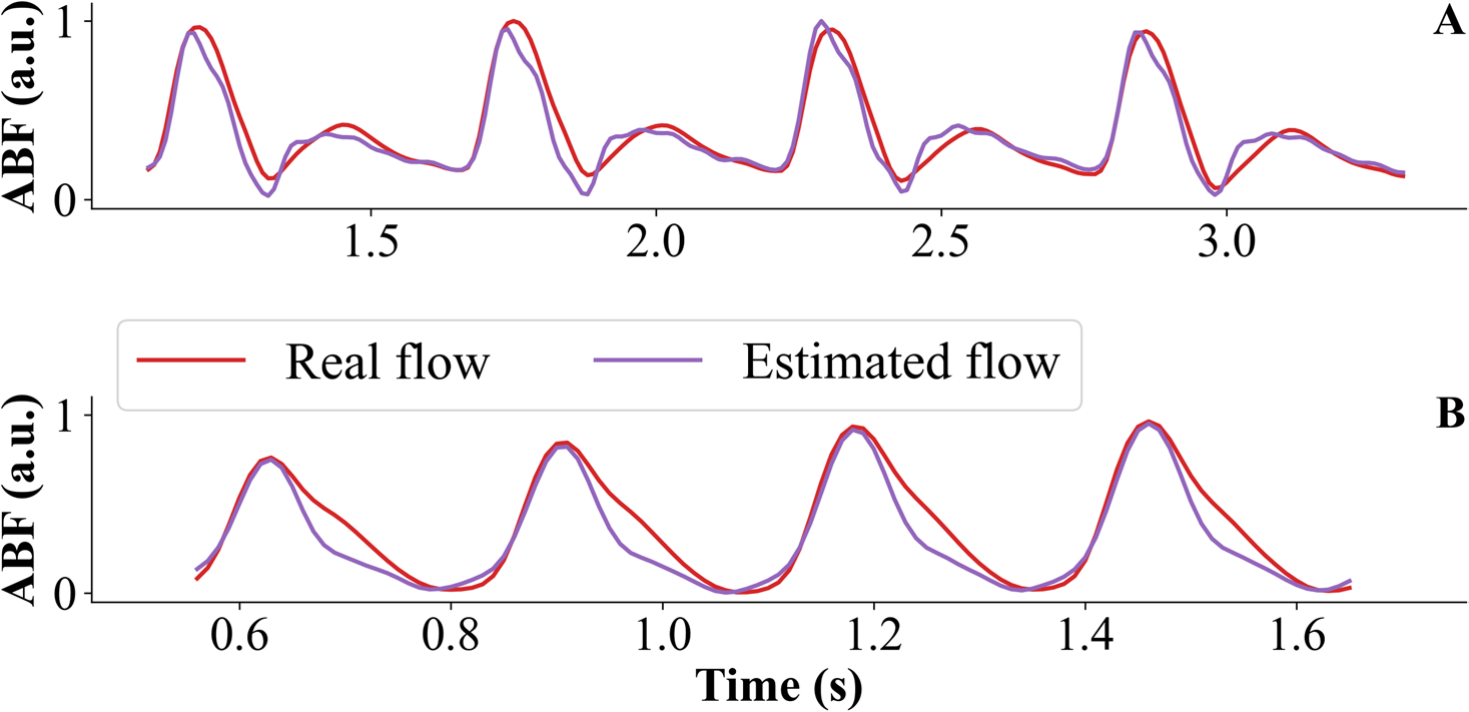
Example of the arterial blood flow (ABF) estimated with the ARX black-box model. The measured ABF normalized is represented with the red line, and the estimated ABF with the purple line. **A** Type A arterial blood pressure (ABP) waveform; **B** Type B ABP waveform

**Fig. 9:**
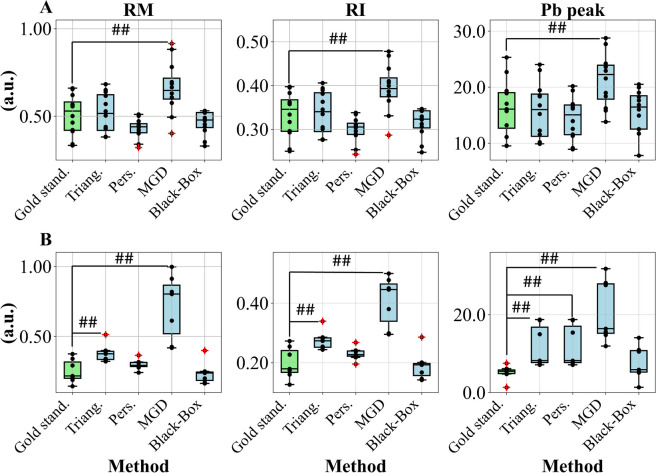
Boxplots representing median (25th, 75th percentile) values of reflection magnitude (RM), reflection index (RI), and amplitude peak of the backward pressure wave (P_b_). **A** Type A waveform. **B** Type B waveform. *Gold stand*., gold standard, the indices were obtained from the measured blood flow; *Triang.*, triangular flow approximation; *Pers.*: personalized flow approximation using the Hermite interpolation function; *MGD*, multi-Gaussian decomposition; *Black-Box*, the proposed black-box modeling for the ABF estimation. Mann–Whitney U test: # *p* < 0.05; ## *p* < 0.01 between the different methods

Figure 8 shows an example of ABF estimation with the ARX model for type A and type B ABP waveforms of the test set. Figure 9 shows the distribution of RM, RI, and P_b_ peak values obtained with all the described methods compared to the gold standard, i.e., obtained using the measured ABF.

For type A waveforms (Fig. 9A), the black-box modeling approach (*Black-Box*) allowed to obtain the most accurate values of the indices together with the triangular flow approximation method (*Triang.*), whereas the personalized flow method (*Pers.*) seems to underestimate the indices, although we did not find any significant difference with respect to the gold standard. On the opposite, the MGD method (*MGD*) showed a significant overestimation. For type B waveforms (Fig. 9B), all methods except for the personalized flow and black-box flow estimation demonstrated a statistically significant overestimation of the values compared to the gold standard (*Gold stand.*). However, the personalized flow method provided a significant overestimation of the peak of the backward wave ${P}_{b}$, hinting that this method is not suitable for the ABP decomposition.

**Fig. 10:**
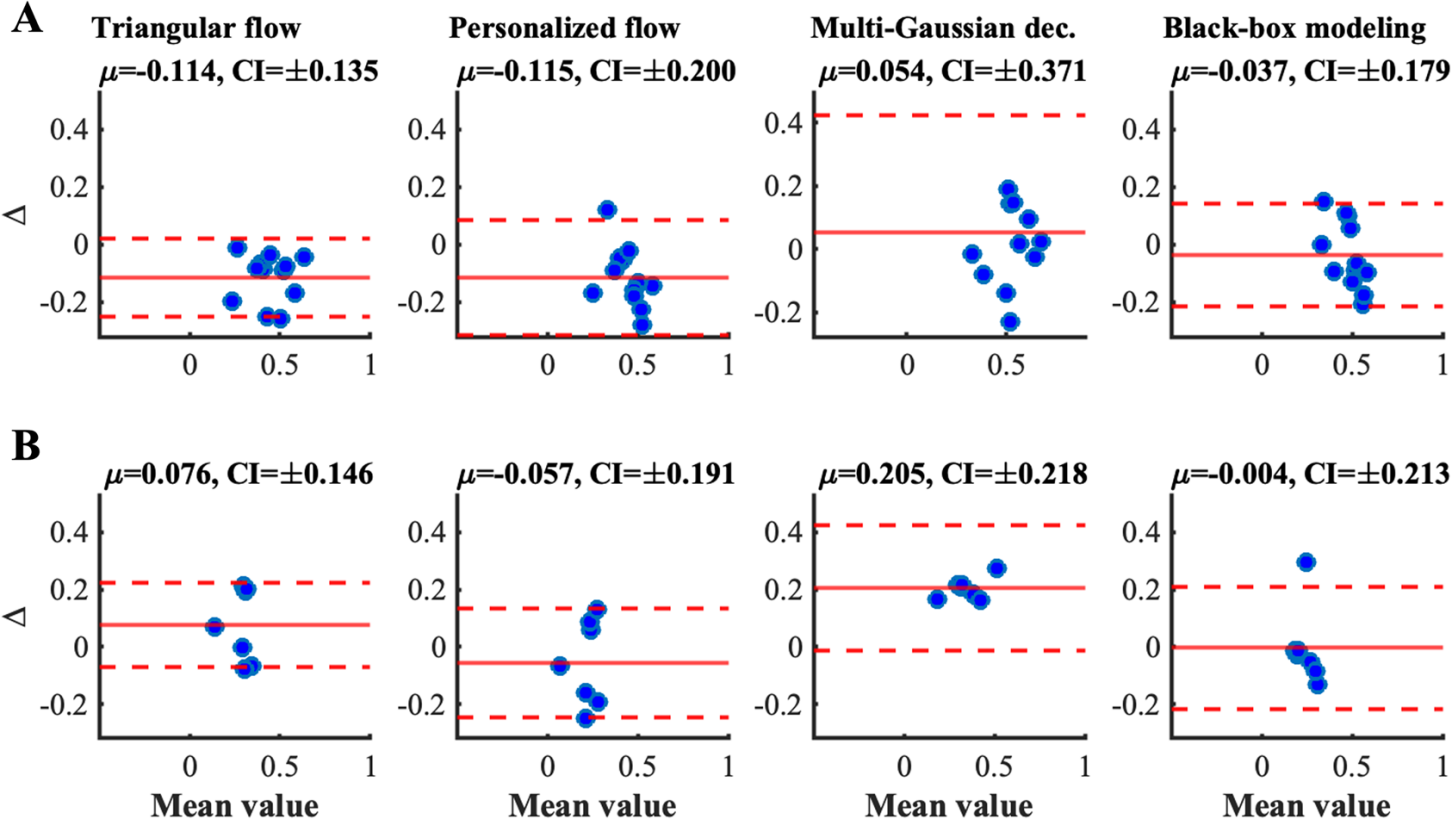
Bland–Altman plot illustrating the values of reflection magnitude (RM) differences between the index computed with the method considered as gold standard and the described method. From left to right, difference between the values obtained with the gold standard and triangular flow estimation, personalized flow estimation, multi-Gaussian decomposition, and black-box estimation. Dashed red line represents 95% confidence intervals (CI) of differences; continuous red line represents the mean value *(μ*) of differences. **A** Type A waveform; **B** Type B waveform

Figure 10 shows the Bland–Altman analyses for the RM index. The lowest bias, i.e., the lowest *μ*, was observed for the black-box approach for both type A and type B. In agreement with the previous results (Fig. 9), the overestimation of MGD in the case of type B waveforms was confirmed.

## Discussion

This study aimed to improve the estimation of PWA indices in a critically ill condition when the ABF is not available. The method proposed for blood flow estimation from the carotid blood pressure consists of a black-box modeling approach; its performance was compared to other methods in terms of indices values representing arterial tree characteristics; in particular, they were compared to those obtained by applying methods like the triangular approximation, the personalized flow estimation, i.e., a modification of the triangular method through a cubic Hermite interpolation to take into account flow concavity, and a multi-Gaussian decomposition method. The latter methods rely all on fiducial points that should be identified on the ABP waveform, i.e., the inflection point and the dicrotic notch; the proposed approach needs instead the measured ABP only, thus avoiding all the uncertainties of a correct identification of these fiducial markers. This can have great advantages especially when the morphological characteristics of the ABP waveform are severely altered.

The triangular method has shown good performance for type A waveforms, but its reliability has been questioned in the case of type B waveforms, which are very common in acute cardiovascular failure, such as after the development of sepsis. We may guess that the incorrect identification of ABP fiducial points by the algorithm may have introduced biases; indeed, in the case of type B waveforms, fiducial points may be difficult to identify even by a visual inspection. The authors originally proposed to set the inflection point at 30% of total ejection time in case of uncertain position [7], but this holds only in physiological conditions, when the heart rate is not excessively high.

The personalized flow estimation is also based on the identification of three fiducial points, which introduces further challenges, especially in the case of type B waveform, when the definition of the intermediate point needs to be adapted, and this could introduce even in this case potential biases. Another possible source of bias could be due to the fact that both of these methods, i.e., triangular and personalized flow, assume zero flow after the dicrotic notch. This is true if blood flow is measured centrally, e.g., in the proximal aorta, but it does not hold anymore if blood flow is measured at peripheral sites, such as in our case for the carotid artery.

The multi-Gaussian decomposition method also requires the identification of the dicrotic notch; moreover, the results highlighted its poor performance, especially in estimating the backward wave, leading to a high P_b_ peak and less accuracy in RM and RI estimation. Our modeling approach instead allowed us to obtain values of wave reflection indices more similar to the gold standard, especially for type B waveforms, where all the other methods dramatically failed to correctly estimate the indices. This type of waveform is typical of a system with little or more diffuse reflections, showing very small secondary rises in systolic pressure, occurring in conditions of severe peripheral vasodilation, such as after administration of vasodilatory drugs (e.g., nitroglycerin) [21] or after development of sepsis, as in our experiment [6]. The availability of a robust methodology for estimating blood flow even in critical cardiovascular conditions would open the possibility of applying WSA techniques in this context and exploring the potential of these indices in the characterization of the cardiovascular status during acute critical illness or hemodynamic stabilization.

Future work should focus on translating these findings to human populations, especially to ICU patients. This involves assessing the performance of the model in patients, considering interindividual variability, and addressing the complexities inherent to more complex clinical conditions where age, comorbidities, and different therapies play an important role. This will help bridge the gap between preclinical research and practical applications in patient care, ensuring the method’s robustness and utility in ICU clinical settings.

### Limitation of the study

The small sample size, especially for the type B dataset, is the major limitation. Further studies are needed to increase the sample size, for example, by considering other experimental critically ill conditions. The model should also be validated in a clinical setting by collecting synchronized and accurate measures of ABP and ABF with echo Doppler for this specific purpose. Finally, although the proposed model is not able to accurately estimate the overall morphology of ABF waveform (Fig. 8), we want to underline that this was not the primary aim of our work, that was, instead, to provide a more reliable tool to better characterize the arterial tree through WSA techniques, especially in critically ill conditions.

## Conclusions

In conclusion, our study presents a novel method for pressure-based blood flow estimation, overcoming challenges in fiducial point identification. The black-box method proves promising, with fewer biases and consistent wave reflection indices for both physiological and non-physiological conditions. However, the small pig population for type B waveforms affects generalizability. While the black-box method shows potential, further validation with a larger sample size is essential.

This research contributes to emphasize ongoing research of arterial blood flow estimation from blood pressure waveform for enhanced applicability of these methodologies in clinical context far from physiological conditions.
